# Dysmenorrhea, with Special Reference to Treatment

**Published:** 1904-04

**Authors:** 


### Dysmenorrhea, with Special Reference to Treatment.

SIMMS, Racine, Wis., (*Medical Herald*), says the clinical diagnosis of dysmenorrhea is in itself easy enough, while the cause is not always so easy to explain. In the cases presented especial attention was paid to the causation of the menstrual pain, as I believe that in this manner I was better able to outline the indications for treatment. It goes without saying that dysmenorrhea from mechanical obstruction is not amenable to medical treatment. Fortunately, however, it has not been as frequent, in my experience, as dysmenorrhea depending upon congestion. The specially disagreeable and intractable form of dysmenorrhea which is accompanied by a fetid discharge as a result of the decomposition of the retained menstrual blood, also comes under discussion here, as the use of douches with antiseptics and deodorants cannot be hoped to affect it permanently, while the employment of more radical medicinal means does bring about the desired effect in this condition.

In congestive dysmenorrhea, and in that form which is accompanied by fetid discharge, the indications are to diminish congestion, by promoting the contractions of the uterus and relieving it of the accumulated blood, to stimulate glandular activity in the mucosa, to restore the tone of the uterus and the nutrition of its tissues to normal, and to relieve spasm and pain.

The following cases illustrate the effects which I obtained with the use of ergoapiol (Smith) in the treatment of dysmenorrhea:

Some months ago I was consulted by a young woman who had suffered from scanty, fetid menstruation, accompanied by a great deal of pain, since the birth of her first child seven years previously. Her labor had been followed by a tear of the perineum which had been left unrepaired, and also a laceration of the cervix uteri. This patient consulted a specialist, but his treatment did not give her relief. Examination revealed the presence of the uterine and perineal lacerations already mentioned, and disclosed a chronic endometritis that had given rise to a fetid discharge and to pain during each menstrual period. I repaired the tears, curetted the uterus, and hoped in this manner to obtain permanent relief of the patient's symptoms. After she had recovered from the operations, she declared that she was feeling better than she had been for years. But very soon the fetid discharge and the pain returned at each menstrual period, and evidently something else had to be done if I wanted to save my reputation. I then tried local applications, alteratives, uterine tonics, etc., all without avail, until finally ergoapiol was given. The result was immediate relief and a gradual and permanent improvement in the menstrual flow until it was free from pain and devoid of any disagreeable odor.

Another type of dysmenorrhea, that which I term "nervous," but which the authorities term "neuralgic," is illustrated by the following case which recently came under my care:

The patient was a young woman who had been married two years, but had not borne any children. She stated that she had pain during the menstrual period from the first onset of menses, and at the time of examination she also complained of a fetid discharge. The menstrual flow was scanty and rarely of blood red color. Just before and after the period she had backache and headache, her complexion was unhealthy, not bright and clear as that of her sister, and she appeared older than she really was. She always dreaded the onset of the menses which recurred with a regularity that is not always present in these cases. She was easily excited, and received impressions vividly and indelibly. Her digestion was poor, and she was often sleepless, irritable, and peevish.

Vaginal examination revealed a slightly thickened os with slight endocervicitis and erosions of the cervix. Cod liver oil, malt extract, hypophosphites, and aromatics, in combination, 25 per cent. of each, were given freely during the intervals between the menstrual periods and for three days before the expected menstruation ergoapiol was given in capsules, one being given three times daily until the discharge ceased. At the fourth period after the beginning of the treatment she was relieved of all her symptoms, and was free from pain and fetor during menstruation. Locally, tincture of iodine and occasionally tampons of ichthyol and glycerine were applied. The cure was permanent and in every way satisfactory.

